# Survey nonresponse among informal caregivers: effects on the presence and magnitude of associations with caregiver burden and satisfaction

**DOI:** 10.1186/s12889-016-2948-6

**Published:** 2016-06-08

**Authors:** Marloes Oldenkamp, Rafael P. M. Wittek, Mariët Hagedoorn, Ronald P. Stolk, Nynke Smidt

**Affiliations:** Department of Epidemiology, University of Groningen, University Medical Center Groningen, PO Box 30.001, Groningen, 9700 RB The Netherlands; Department of Sociology, University of Groningen, Grote Rozenstraat 31, Groningen, 9712 TG The Netherlands; Department of Health Sciences, Health Psychology, University of Groningen, University Medical Center Groningen, PO Box 30.001, Groningen, 9700 RB The Netherlands; Healthy Ageing, Population and Society, HAPS, University of Groningen, Groningen, The Netherlands

**Keywords:** Nonconsent bias, Nonresponse bias, Informal care, Research participation, Caregiving research

## Abstract

**Background:**

Informal caregiving is becoming more relevant with current trends such as population ageing. However, little is known about nonconsent and nonresponse bias in caregiving research. We investigated nonconsent and nonresponse bias in a sample of informal caregivers who participated in the LifeLines Cohort Study, and were invited for participation in an additional caregiving study.

**Methods:**

We compared socio-demographic characteristics, caregiver health, caregiving situation, and caregiver outcomes of nonconsent and consent caregivers, and nonresponse and response caregivers, on LifeLines data, by using Chi-square tests, Independent Sample T-tests, and Mann-Whitney tests. Furthermore, we examined the influence of nonconsent and nonresponse on the presence and magnitude of the associations between caregiver characteristics and two commonly used caregiving outcomes (caregiver burden and satisfaction). We conducted multinomial logistic regression analyses, including interaction terms with nonconsent and nonresponse.

**Results:**

Within a subcohort of 8443 caregivers, aged >18 years, 5095 caregivers (60 %) gave consent for participation in the caregiving study. Within the subgroup of 2002 caregivers who received the questionnaire, 965 (48 %) responded. Caregivers who were highly involved in caregiving (i.e. high time investment, high caregiver burden), gave more commonly consent to participate, and responded more often to the questionnaire. Nonconsent and nonresponse influenced the associations between caregiver characteristics and caregiver burden for only a few characteristics, mainly indicating the level of caregiving involvement (e.g. time investment, caregiving duration). Especially for caregiver burden, these indicators were stronger for consent and response caregivers than for nonconsent and nonresponse caregivers.

**Conclusions:**

Our findings are important for caregiving research, as they emphasized that participation might not be evenly distributed among caregivers, and that the possibility of nonconsent and nonresponse bias should be considered.

**Electronic supplementary material:**

The online version of this article (doi:10.1186/s12889-016-2948-6) contains supplementary material, which is available to authorized users.

## Background

In population-based cohort studies using mailed questionnaires, the presence of nonresponse is inevitable [1, 2]. When the likelihood to respond is related with key exposures, outcomes, or associations, nonresponse bias is introduced into the study [1, 2]. Although many researchers use the nonresponse rate itself as an indicator of nonresponse bias, the relation between the likelihood to respond, relevant exposures, and outcomes is most important for the presence and magnitude of nonresponse bias [1–3]. Furthermore, a thorough evaluation of nonresponse bias not only consists of a study of the bias in exposures and outcomes, but also of the bias in associations between exposures and outcomes [4]. Nonresponse bias might challenge the external validity of the study results, as it negatively affects the generalizability of the results to the target population [1, 2]. In addition to nonresponse bias, study results might also be biased due to nonconsent. Nonconsent and nonresponse should be considered as two different constructs, because not all individuals who give consent for participation in research respond [5]. Consent might not be evenly distributed among individuals, which could affect the external validity of study results [6]. Studying both nonconsent and nonresponse provides an understanding of the stages of the recruitment process in which bias might occur.

With current trends such as population ageing, caregiving research aiming to improve our understanding of caregiving experiences and outcomes of informal caregiving, is becoming more relevant [7]. However, only little is known about nonconsent and nonresponse of informal caregivers in caregiving research. One population-based study has suggested that being an informal caregiver, compared to not being an informal caregiver, is related to higher nonresponse [8], but questions on whether and which characteristics of the caregiving situation itself relate to nonconsent and nonresponse remain largely unanswered. This can mainly be explained by the lack of information about nonrespondents, which hinders the assessment of nonresponse and nonresponse bias [2, 9].

On the one hand, research participation requires time and energy, and this time and energy adds to the time and energy necessary for informal caregiving [10]. This might in particular make caregivers who spent a lot of time on their caregiving, or who experience high burden, more inclined to reject research participation [11, 12]. On the other hand, research has also suggested that people are more likely to participate in research when the research topic is highly relevant to their own life [13]. From this perspective, we expect higher consent and response rates among caregivers with a high time investment and caregiver burden, since their role as caregiver constitutes a major part of their lives. In addition, caregivers have reported positive effects from participation in qualitative research, because it offers them an opportunity to talk about their experiences [14, 15]. Although our study is a quantitative study, caregivers might also be able to display their experiences and thoughts in a questionnaire. Research participation might then function as an opportunity for alleviation or relieve, with highest consent and response rates for caregivers who experience a high burden.

To expand our knowledge on nonconsent and nonresponse in caregiving research, the main objective of this study was to evaluate the nonconsent bias and nonresponse bias in a questionnaire about informal caregiving, in a sample of informal caregivers in the LifeLines Cohort Study [16, 17]. It should be noted that prior to giving consent and responding to the informal care questionnaire, the informal caregivers already decided to participate in LifeLines. Thus, we evaluated nonconsent and nonresponse in a sample of informal caregivers who might have a more positive attitude towards research participation than informal caregivers in general.

Firstly, we studied the differences between consent and nonconsent caregivers, and between response and nonresponse caregivers, with regard to their socio-demographic characteristics, caregiver health, caregiving situation, and the caregiving outcomes burden and satisfaction, which are two commonly used outcomes in caregiving research (see for example [18, 19]). Secondly, we studied the influence of nonconsent and nonresponse on the presence and magnitude of the associations between the various characteristics and caregiving outcomes (caregiver burden and caregiver satisfaction).

## Methods

### The Lifelines Cohort Study

LifeLines is a multi-disciplinary prospective population-based cohort study examining in a unique three-generation design the health and health-related behaviours of 167,729 persons living in the North of the Netherlands. The study profile of LifeLines is described elsewhere [17]. Briefly, LifeLines employs a broad range of investigative procedures in assessing the biomedical, socio-demographic, behavioural, physical and psychological factors which contribute to the health and disease of the general population, with a special focus on multimorbidity and complex genetics. The LifeLines Cohort Study is approved by the medical ethical committee of the University Medical Center Groningen, the Netherlands. The data collection started in December 2006. In December 2013, the last participant was included in the cohort, and the cohort will be followed for at least 30 years. All participants signed an informed consent form before they received an invitation for the first comprehensive physical examination and baseline questionnaire. Participants are invited for a renewed physical examination every 5 years, and they receive extensive follow-up questionnaires every 1,5 year. Currently, 3 follow-up questionnaires are running, and since January 2014 participants are invited again for the second physical examination at one of the LifeLines research sites.

### Subcohort of informal caregivers in the LifeLines Cohort Study

A subcohort of informal caregivers in LifeLines was defined, using the second follow up questionnaire, which is distributed among all LifeLines participants aged 18 years and older. This questionnaire includes, besides many other topics, a question for identification of informal caregivers, eight questions about the caregiving situation, and a question for consent for participation in an additional informal care questionnaire. Informal care was described to the LifeLines participants as follows: ‘*It is unpaid care, because of chronic disabilities and/or health problems. Informal care concerns care for a loved one, for example your partner, a family member, friend, or other relative. Voluntary work and care for healthy children is not included’* (see also [20]). Participants who indicated to provide informal care were considered as informal caregivers, and they answered several basic questions about their caregiving situation. In addition, they were asked for their consent for participation in an additional informal care questionnaire. Informal caregivers who gave their consent received, within 2 weeks after completion of the second follow up questionnaire, this informal care questionnaire by post (paper questionnaire) or email (digital questionnaire). They were requested to fill out the questionnaire and return it, using an enclosed reply envelope for the paper questionnaires. Because of organizational and financial reasons, no reminders were sent for the informal care questionnaire. Although the use of reminders can be an effective way to increase response rates [21, 22], a higher response rate does not necessarily decrease nonresponse bias [2, 3]. An overview of all the data collected in LifeLines, including the questions about informal care, is provided in the online open access LifeLines Data Catalogue [20].

### Variables

#### Consent and response

In the second follow up questionnaire of LifeLines, we distinguished caregivers who gave their consent, and those who did not give this consent (0 = nonconsent, 1 = consent) for participation in the additional informal care questionnaire. Subsequently, based on the informal care questionnaire, we differentiated between consent caregivers who returned and not returned the informal care questionnaire (0 = nonresponse, 1 = response).

#### Socio-demographic characteristics

Socio-demographic characteristics concerned age, sex, partner status (yes/no), household composition, educational level, employment status (employed/unemployed), and voluntary work (yes/no). Household composition consisted of the number of people living in the household (1/2/3/4/5 and more), and whether one had children aged 0 to 12 years (yes/no) [23]. The highest educational level was categorized into primary, secondary, and tertiary education, according to the Standard education classification of Statistics Netherlands (Standaard onderwijs indeling 2006, edition 2013/2014).

#### Caregiver health

Caregiver health contained the caregiver’s general health perception and level of somatisation. General health perception was assessed using the first question of the RAND-36: ‘In general, would you say your health is: excellent, very good, good, fair, or poor?’ [24]. This is a feasible, inclusive, and informative measure of health status, and has been shown to be an important and robust predictor of multiple future health outcomes [25, 26]. Because of very low numbers in the category poor (0.6 %), we used the following categories: (0) poor/fair, (1) good, (2) very good (3) excellent. The level of somatisation, which is the reflection of psychological distress in physical symptoms, was measured with the somatisation subscale of the SCL-90 (Symptom Check List) [27]. This somatisation subscale is a sum score of 12 items on the presence of physical symptoms like headaches, nausea, or dizziness, ranging from 12 to 60 (Cronbach’s alpha 0.80), with a higher score indicating more somatisation symptoms. Missing data on separate items of the somatisation subscale were imputed using corrected item mean substitution, only for the caregivers with at least half of the items complete (≥6 valid items) [28].

#### Caregiving situation

The caregiving situation was characterized by the following factors: (a) the type of care relationship between caregiver and care recipient, which included whether caregivers cared for their spouse (yes/no), their parent (in-law) (yes/no), their child (in-law) (yes/no), or someone else (yes/no), (b) whether caregivers cared for more than one care recipient (yes/no), (c) whether caregivers lived together with their care recipient (yes/no), (d) the duration of providing informal care, measured in years, and (e) the time investment in caregiving. For the type of care relationship, we combined caregivers of parents and parents in-law, because of low numbers of caregivers of parents in-law. In addition, in a large meta-analysis few differences emerged between caregivers of parents and of parents in-law [29]. For time investment in caregiving we distinguished three different types of care: household care (i.e. cleaning the house, preparing food and drinks, shopping for groceries), personal care (i.e. helping with dressing and undressing, washing, eating, administering medication), and other care (i.e. helping and accompanying with outdoor activities such as family visits/contacts with GP, arranging assistance, organizing financial/administrative matters). Caregivers retrospectively indicated the hours a week spent on each type of care. This is a valid method to measure time investment in caregiving, provided that it is taken into account that caregivers might overestimate their time investment. They might have difficulties with the distinction between caregiving and non-caregiving related tasks, and with the simultaneous performance of multiple caregiving tasks [30]. Because of positively skewed distributions, we categorized the hours per care-related task into 0 h, 1–4 h, 4–8 h, and >8 h, in accordance with the duration of an average (half) working day [23].

#### Caregiving outcomes

Caregiver burden experienced by caregivers was measured by asking how difficult or burdening the caregiving was, on a scale from 0 (not difficult at all/minimal burden), to 100 (far too difficult/severe burden). This single question is based on the self-rated burden scale (SRB), and is a concise and simple measure of a caregiver’s overall burden, based on a caregiver’s own assessment [31, 32]. The SRB has shown to be a feasible, reliable, and valid measurement of caregiver burden in a wide range of caregivers [31, 32]. Comparable to caregiver burden, the degree of satisfaction derived from caregiving was measured by asking caregivers how much satisfaction they derive from their caregiving, also on a scale from 0 (no satisfaction) to 100 (much satisfaction).

### Statistical analyses

Firstly, we described the socio-demographic characteristics, caregiver health, caregiving situation, and caregiving outcomes for nonconsent caregivers, consent caregivers, nonresponse caregivers, and response caregivers, separately. Differences between nonconsent and consent caregivers, and between nonresponse and response caregivers, were tested using Chi-square tests (categorical variables), Independent Sample T-tests (normally distributed continuous variables), and Mann-Whitney tests (not normally distributed continuous variables). Secondly, we evaluated the influence of nonconsent and nonresponse on the presence and magnitude of the associations between the caregiver characteristics (socio-demographic, health, situation) and the two caregiver outcomes. The outcomes caregiver burden and caregiver satisfaction were not normally distributed, therefore we used tertiles of caregiver burden and caregiver satisfaction (low, medium, high). For each independent variable, we estimated a separate multinomial logistic regression model, including the variable itself, the variable consent no/yes or response no/yes, and the interaction term of the two variables (variable * consent no/yes, variable * response no/yes). By estimating a model with consent coded as 0 = nonconsent and 1 = consent , and a model with consent coded as 0 = consent and 1 = nonconsent, we obtained the odds ratio’s and 95 % confidence intervals for both consent and nonconsent caregivers. The same approach was used for response. A significant interaction term (*p* ≤ .05) indicated the presence of nonconsent bias or nonresponse bias. In the result section, only the variables with a significant interaction term are presented.

In order to prevent the loss of information and potential bias due to selective refusal to answer questions, we dealt with item nonresponse on the hours of household care (8.2 % missing values), hours of personal care (14.7 % missing values), and hours of other care (4.0 % missing values), by using multiple imputation (Fully Conditional Specification; 10 imputations; predictive mean matching as model type for scale variables). Rubin’s Rules were applied for pooling the results that concerned the hours of household, personal, and other care [33, 34]. Sensitivity analyses in which we used the non-imputed data for hours of household care, personal care, and other care, did not provide substantial different results. All statistical analyses, including the multiple imputation, were performed using IBM SPSS Statistics (version 22).

## Results

### Recruitment of informal caregivers

**Fig. 1:**
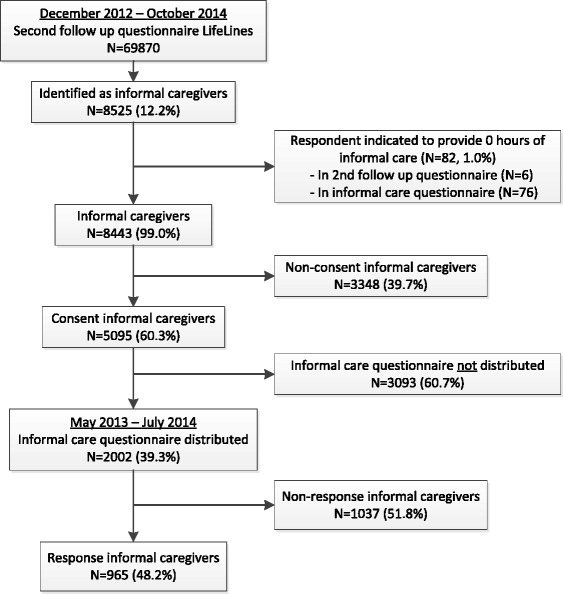
Flow chart of informal caregivers in the LifeLines cohort study

Between December 2012 and October 2014, 69,870 LifeLines participants participated in the second follow up questionnaire of LifeLines, and 8443 informal caregivers were identified (Fig. 1). Of those 8443 informal caregivers, 60.3 % gave consent (*N* = 5095) for participation in the informal care questionnaire. Due to logistical and financial reasons, the informal care questionnaire was only distributed among the LifeLines participants who were identified as caregivers and gave consent between May 2013 and July 2014 (39.3 % of all consent caregivers; *N* = 2002). The period of May 2013 – July 2014 included a whole year, so that seasonal effects could be excluded. Of all 2002 consent caregivers who received the informal care questionnaire, 965 returned this questionnaire, resulting in a response rate of 48.2 %.

### Consent and nonconsent caregivers

The characteristics of the total group of caregivers (*N* = 8443), the nonconsent caregivers (*N* = 3348) and the consent caregivers (*N* = 5095) are presented in Table 1. Overall, 60.3 % of the caregivers (*N* = 5095) gave consent for the additional informal care questionnaire. Compared with nonconsent caregivers, consent caregivers had less often a partner or primary education, and were more often employed or doing voluntary work. In addition, consent caregivers cared more often for a child (in-law) or for more than 1 care recipient, and they lived more often together with their care recipient. They cared on average for a longer duration, provided more hours of household care, personal care, and other care, but they also experienced, on average, more burden from their caregiving, and derived more satisfaction from their caregiving. No significant differences were found with regard to caregiver health.

**Table 1:** Characteristics of all subgroups of caregivers

	All identified caregivers				All consent caregivers who received the additional caregiving questionnaire			
	Total	Nonconsent caregivers	Consent caregivers	*p* ^c^	Total	Nonresponse caregivers	Response caregivers	*p* ^c^
Total (N, %)	8443 (100 %)^a^	3348 (39.7 %)^a^	5095 (60.3 %)^a^		2002 (39.3 %)^a^	1037 (51.8 %)^a^	965 (48.2 %)^a^	
Socio-demographic characteristics								
Age, mean (SD)	52.2 (9.68)	52.3 (10.12)	52.1 (9.39)	.566	52.3 (9.94)	51.6 (9.98)	53.1 (9.85)	.001
Female	75.9 %	75.9 %	75.9 %	.998	75.0 %	74.8 %	75.1 %	.878
Partner, yes	88.6 %	89.9 %	87.8 %	.004	87.9 %	88.4 %	87.3 %	.462
Nr. of people in household, mean (SD)	2.7 (1.15)	2.7 (1.13)	2.7 (1.16)	.968	2.7 (1.17)	2.8 (1.18)	2.7 (1.15)	.023
Children aged 0–12, yes	16.1 %	16.3 %	16.0 %	.694	16.3 %	17.8 %	14.6 %	.051
Educational level				.000				.038
Primary	30.9 %	34.8 %	28.2 %		27.8 %	25.8 %	30.0 %	
Secondary	41.2 %	39.1 %	42.5 %		41.4 %	43.9 %	38.7 %	
Tertiary	27.9 %	25.9 %	29.2 %		30.8 %	30.4 %	31.3 %	
Employed, yes	71.7 %	69.2 %	73.2 %	.000	72.5 %	75.9 %	68.8 %	.000
Voluntary work, yes	36.9 %	32.1 %	40.1 %	.000	40.5 %	42.2 %	38.5 %	.094
Caregiver health								
General health perception				.640				.343
Poor/fair	13.3 %	13.5 %	13.2 %		13.7 %	12.5 %	15.0 %	
Good	60.2 %	60.8 %	59.9 %		59.4 %	61.0 %	57.6 %	
Very good	21.2 %	20.6 %	21.7 %		21.0 %	20.6 %	21.5 %	
Excellent	5.2 %	5.1 %	5.3 %		5.9 %	5.9 %	5.9 %	
Somatisation, median (IQ range)	16 (14–19)	16 (14–19)	16 (14–19)	.799	16 (14–19)	16 (14–19)	15 (14–19)	.025
Caregiving situation								
Caregiver cares for:								
Spouse, yes (vs. no)	11.1 %	11.0 %	11.2 %	.744	11.7 %	9.4 %	14.2 %	.001
Parent (in-law), yes (vs. no)	63.5 %	62.6 %	64.0 %	.199	62.9 %	63.7 %	62.0 %	.421
Child (in-law), yes (vs. no)	14.8 %	13.5 %	15.7 %	.006	15.5 %	14.5 %	16.7 %	.174
Someone else, yes (vs. no)	24.3 %	24.9 %	23.9 %	.289	24.8 %	27.7 %	21.8 %	.002
More than 1 care recipient, yes	28.0 %	26.0 %	29.3 %	.001	29.7 %	31.5 %	27.9 %	.079
Living together with care recipient, yes	20.5 %	18.9 %	21.6 %	.004	21.5 %	18.4 %	24.8 %	.001
Caregiving duration (years), median (IQ range)	4 (2–10)	4 (1–10)	5 (2–10)	.000	4 (2–10)	4 (2–10)	4 (2–10)	.812
Hours of household care tasks^b^				.000				.015
0 h	22.0 %	23.5 %	21.0 %		20.8 %	21.7 %	19.8 %	
1–4 h	55.6 %	56.4 %	55.0 %		54.5 %	56.0 %	52.8 %	
4–8 h	11.2 %	10.5 %	11.7 %		11.8 %	11.7 %	12.0 %	
> 8 h	11.2 %	9.6 %	12.3 %		12.9 %	10.6 %	15.3 %	
Hours of personal care tasks^b^				.000				.375
0 h	61.6 %	65.5 %	59.0 %		59.3 %	59.7 %	59.0 %	
1–4 h	27.9 %	25.7 %	29.2 %		29.1 %	30.0 %	28.3 %	
4–8 h	5.3 %	4.4 %	5.9 %		5.5 %	4.9 %	6.1 %	
> 8 h	5.3 %	4.4 %	5.9 %		6.0 %	5.4 %	6.6 %	
Hours of other care tasks^b^				.000				.000
0 h	9.9 %	11.7 %	8.7 %		9.4 %	11.3 %	7.5 %	
1–4 h	69.5 %	71.1 %	68.5 %		67.9 %	69.5 %	66.1 %	
4–8 h	13.1 %	11.5 %	14.2 %		13.8 %	12.3 %	15.2 %	
> 8 h	7.5 %	5.7 %	8.7 %		8.9 %	6.8 %	11.2 %	
Caregiving outcomes								
Caregiver burden, median (IQ range)	10 (4–40)	10 (2–36)	20 (5–50)	.000	20 (5–50)	11 (5–40)	20 (5–50)	.007
Caregiver satisfaction, median (IQ range)	80 (50–90)	75 (50–90)	80 (50–90)	.000	80 (50–90)	80 (50–90)	80 (50–90)	.254

^a^Number of respondents might vary between variables due to item non-response

^b^Item non-response for hours of household care, personal care, and other care was imputed using multiple imputation

^c^Chi-square test is reported for all variables, except for age and nr. of people in household (Independent Sample T-test) and for somatization, caregiving duration, caregiver burden, and caregiver satisfaction (Mann-Whitney test)

### Selection of consent caregivers for additional informal care questionnaire

The informal care questionnaire was only distributed among the informal caregivers who gave consent for participation in the period May 2013 – July 2014 (39.3 % of all consent caregivers, *N* = 2002). To test whether this was a selective group, we compared this group with all consent caregivers who did not receive the informal care questionnaire because they gave consent for participation outside the period May 2013 – July 2014 (60.7 % of all consent caregivers, *N* = 3093). The two subsets of consent caregivers did not statistically significantly differ on socio-demographic characteristics, caregiving situation, caregiver health, and caregiving outcomes (see Additional file 1).

### Response and nonresponse caregivers

The characteristics of the consent caregivers who received the informal care questionnaire (*N* = 2002), the nonresponse caregivers (*N* = 1037), and response caregivers (*N* = 965) are presented in Table 1.

Overall, 48.2 % of the consent caregivers responded to the informal care questionnaire. Compared with nonresponse caregivers, response caregivers were on average older, were living with less other persons in their household, had more often primary education, were less often employed, and had on average lower levels of somatisation. Furthermore, response caregivers were more often caring for their spouse, and were more often living together with their care recipient. They provided more hours of household care and more hours of other care, and experienced, on average, a higher burden from their caregiving.

### Nonconsent bias

In Table 2, the associations between the independent variables (i.e. socio-demographic characteristics, caregiver health, caregiver situation) and both caregiver outcomes are presented for nonconsent and consent caregivers (see columns nonconsent and consent). The last column presents the interaction terms of the independent variables with consent no/yes (variable * consent no/yes). Only the results with significant interaction terms (*p* ≤ .05) are presented. In general, the associations between the independent variables and caregiver burden were more evident in the consent group than in the nonconsent group of caregivers. A longer caregiving duration, lower caregiver age, and 1–4 h of household care provision (vs. 0 h) were statistically significantly related to higher caregiver burden in the consent group, but not in the nonconsent group. Living together with the care recipient and providing >8 h of household care (vs. 0 h) were statistically significantly related to higher caregiver burden for both nonconsent and consent caregivers, but the associations were stronger in the consent group. For the outcome caregiver satisfaction, only few differences emerged. Being female was statistically significantly related to lower satisfaction in the consent group, but not in the nonconsent group. In contrast, providing 4–8 h personal care (vs. 0 h) and caring for a parent were statistically significantly related to lower satisfaction in the nonconsent group, but not in the consent group.

**Table 2:** Influence of nonconsent on presence and magnitude of associations with caregiver burden and caregiver satisfaction^a^

	Nonconsent		Consent		Interaction		
	(*N* = 3348)		(*N* = 5095)		(variable * consent)		
	OR	(95 % CI)	OR	(95 % CI)	OR	(95 % CI)	*p*
**Caregiver burden**							
Medium burden vs. low burden							
Caregiving duration	1.00	(.99–1.01)	1.02	(1.01–1.03)	1.02	(1.01–1.04)	.009
High burden vs. low burden							
Age	.99	(.99–1.00)	.98	(.98–.99)	.99	(.98–1.00)	.049
Caring for spouse	1.22	(.93–1.61)	1.83	(.99–3.40)	1.50	(1.06–2.12)	.024
Living together with care recipient	1.91	(1.55–2.37)	2.68	(2.25–3.19)	1.40	(1.06–1.84)	.017
1–4 h household care (vs. 0 h)	.97	(.78–1.20)	1.31	(1.10–1.56)	1.36	(1.03–1.79)	.032
>8 h household care (vs. 0 h)	2.15	(1.54–2.99)	4.42	(3.34–5.85)	2.06	(1.34–3.17)	.001
**Caregiver satisfaction**							
Medium satisfaction vs. low satisfaction							
Female	1.05	(.87–1.28)	.73	(.62–.86)	.70	(.54–.90)	.006
4–8 h personal care (vs. 0 h)	.55	(.32–.95)	1.08	(.78–1.49)	1.95	(1.00–3.77)	.052
High satisfaction vs. low satisfaction							
Female	1.01	(.83–1.23)	.78	(.66–.92)	.77	(.60–1.00)	.046
Caring for parent	.74	(.62–.87)	.93	(.93–1.07)	1.27	(1.01–1.58)	.040

*OR* Odds Ratio, *95* % *CI* 95 % confidence interval

ORs are based on multinomial logistic regression models, each containing the specific variable itself, the variable consent yes/no, and the interaction term of the two variables

^a^Only statistically significant interactions with consent yes/no are presented (*p* < .05)

### Nonresponse bias

In Table 3 the associations that differed between nonresponse and response caregivers are presented, similar to the way in which we presented the results in Table 2. With regard to outcome caregiver burden, we found that being female was statistically significantly related to higher caregiver burden in the nonresponse group, but not in the response group. On the contrary, caring for a spouse was statistically significantly related to higher burden in the response group, but not in the nonresponse group. Both nonresponse and response caregivers experienced higher caregiver burden when they lived together with their care recipient, but this association was stronger for response caregivers. The only difference in associations for outcome caregiver satisfaction was that doing voluntary work related to higher satisfaction in the nonresponse group, but not in the response group.

**Table 3:** Influence of nonresponse on presence and magnitude of associations with caregiver burden and caregiver satisfaction^a^

	Nonresponse		Response		Interaction		
	(*N* = 1037)		(*N* = 965)		(variable * response)		
	OR	(95 % CI)	OR	(95 % CI)	OR	(95 % CI)	*p*
**Caregiver burden**							
Medium burden vs. low burden							
Voluntary work	1.27	(.93–1.73)	.78	(.55–1.10)	.62	(.39–.98)	.039
High burden vs. low burden							
Female	1.84	(1.28–2.64)	1.09	(.75–1.56)	.59	(.35–.99)	.044
Caring for spouse	1.22	(.73–2.03)	2.56	(1.56–4.19)	2.10	(1.03–4.26)	.041
Living together with care recipient	2.10	(1.40–3.15)	4.07	(2.64–6.26)	1.94	(1.07–3.50)	.029
**Caregiver satisfaction**							
High satisfaction vs. low satisfaction							
Voluntary work	1.41	1.04–1.93	.74	.53–1.04	.53	(.33–.83)	.005

*OR* Odds Ratio, *95* % *CI* 95 % confidence interval

ORs are based on multinomial logistic regression models, each containing the specific variable itself, the variable response yes/no, and the interaction term of the two variables

^a^Only statistically significant interactions with response yes/no are presented (*p* < .05)

## Discussion

Given the ageing of the population [7], research that improves our understanding of caregiving experiences and outcomes of informal caregiving is becoming more relevant. Only little is known about selective participation due to nonconsent and nonresponse in caregiving research. In this study, we evaluated the nonconsent and nonresponse in a sample of informal caregivers in the LifeLines Cohort Study [16, 17], and studied to what extent the nonconsent and nonresponse influenced the presence and magnitude of associations with caregiver burden and caregiver satisfaction. We found several, but small (<8 %), differences between nonconsent and consent caregivers, and between nonresponse and response caregivers. Overall, consent and response caregivers more often had a high involvement in caregiving (e.g. high time investment, high caregiver burden), compared with nonconsent and nonresponse caregivers. This is in line with previous research [6, 13], indicating that individuals are more inclined to participate in a study when the research topic is highly relevant to their own life. A high involvement in caregiving might not so much be a constraint for research participation, as has been suggested by some caregiving studies [10–12], but it might be more an indication of the extent to which caregiving constitutes a relevant part of one’s life. Next to that, the opportunity to talk about caregiving experiences has been pointed out by caregivers as a positive effect of participation in qualitative research [14, 15], and a high involvement in caregiving might strengthen the needs to express these experiences in a quantitative study as well. Differences on socio-demographic and health characteristics were largely in line with studies with a comparable study design (i.e. participants of an existing cohort study being invited for participation in an additional study) [5, 8, 35, 36], except for educational level. Lower educated caregivers were less likely to give consent, but they were more likely to respond to this caregiving questionnaire. This corresponds with the idea that nonconsent and nonresponse are two different constructs [5].

Nonconsent and nonresponse influenced the associations between caregiver characteristics and caregiver burden for only a few characteristics, mostly indicators of the level of caregiving involvement (i.e. time investment, caregiving duration). In general, a high involvement in caregiving appeared to be stronger related to caregiver burden for consent and response caregivers than for nonconsent and nonresponse caregivers. The associations between caregiver characteristics and caregiver satisfaction were less affected by nonconsent and nonresponse. Although nonresponse does not necessarily cause bias in associations between relevant exposures and outcomes in other areas of healthcare research (see for example [4, 5, 35]), our results indicate that in caregiving research some associations with caregiver burden may be biased due to nonconsent and nonresponse. Therefore, an important consideration for caregiving research is that caregiving samples might not only be overrepresented by highly involved caregivers, but that this also may result in biased associations with caregiver burden.

Our study has some limitations that need to be mentioned. First, our response rate of 48 % was lower than the response rate of 68 % for the second follow up questionnaire of LifeLines. Half of the response to the second follow up questionnaire of LifeLines (34 %) is achieved after reminders are sent, which might explain the differences in response rates. Unfortunately, because of logistical and financial reasons, no reminders were sent for the informal care questionnaire. This is a limitation of the study, as it might have introduced selection bias. Response rates in other caregiving studies varied from, for example, 31 % [37], to 81–96 % [38]. As selection criteria, recruitment methods, and also the content of the research projects differ between studies, it is difficult to explain the large differences in response rates [39].

Second, an inevitable consequence of the LifeLines study design and the set-up of our caregiving study within LifeLines, is that all caregivers were already participating in LifeLines when they decided to participate in the informal care questionnaire. Previous research did not find other mechanisms to be operating for this second-stage nonresponse, in comparison to initial nonresponse [5, 36], so an underrepresentation of a specific group of respondents due to initial nonresponse might be reinforced by second-stage nonresponse [36]. LifeLines has shown to be broadly representative for the Northern part of the Netherlands [40], but we do not know to what extent caregivers in the LifeLines population are representative for all caregivers in the Northern part of the Netherlands. Although the prevalence of informal care in the Dutch adult population depends on how informal care is defined, the prevalence of 12 % of informal caregivers in the LifeLines population is relatively low, compared with caregiver prevalence in other Dutch studies (15–20 %) [41]. For participation in a large and long-term study like LifeLines, with healthy ageing as the general research topic, a high involvement in caregiving might not play a role at all, or might even constrain participation. Therefore, it should be taken into account that we cannot rule out the presence of a nonresponse bias in the initial participation in the LifeLines Cohort Study.

Third, we have distributed the informal care questionnaire only between May 2013 and July 2014. Although consent caregivers receiving and consent caregivers not receiving the questionnaire did not differ on included characteristics, the possibility of selection dependent on non-observed characteristics still exists. And fourth, information about the health situation of the care recipient and the availability of (in)formal support was only available for response caregivers and therefore could not be studied. However, the care recipient’s health situation and (in)formal support may be related to a caregiver’s decision about research participation. For example, it has been shown that a more advanced dementia stage and personality changes in the care recipient relate to a higher nonresponse [42].

## Conclusions

We found that caregivers who were highly involved in their caregiving (i.e. high time investment, high burden) more often gave consent and responded to the informal care questionnaire, and that the associations between some indicators of the level of caregiving involvement and caregiver burden were stronger for consent and response caregivers. This information is important for caregiving research, because it emphasizes that not all caregivers will participate in caregiving studies, that participation might not be evenly distributed among caregivers, and that some associations with caregiver burden might be overestimated. Therefore, researchers should carefully investigate the potential impact of nonconsent and nonresponse bias for the interpretation of their study results. In the design of new caregiving studies, researchers may consider options to obtain additional information about nonconsent and nonresponse caregivers. Such information will facilitate a better understanding of the extent to which study results might be biased due to nonconsent and nonresponse.

## Additional file

Additional file 1: Table S1. Characteristics of consent caregivers receiving and consent caregivers not receiving informal care questionnaire. (DOCX 19 kb)
