# Supplementary material for: Survey nonresponse among informal caregivers: effects on the presence and magnitude of associations with caregiver burden and satisfaction
Source: BMC Public Health. 2016 Jun 8;16:480. doi: 10.1186/s12889-016-2948-6 (PMC4898385; doi:10.1186/s12889-016-2948-6)
Supplement: Additional file 1: Table S1. — Characteristics of consent caregivers receiving and consent caregivers not receiving informal care questionnaire. (DOCX 19 kb) [file 12889_2016_2948_MOESM1_ESM.docx]

| Table S1: Characteristics of consent caregivers receiving and consent caregivers not receiving informal care questionnaire | | | |
| --- | --- | --- | --- |
|  | Consent caregivers (N=5095, 100%) | |  |
|  | Caregiving questionnaire received | |  |
|  | No | Yes | p ^c^ |
| Total (N, %) | 3093 (60.7%) ^a^ | 2002 (39.3%) ^a^ |  |
| Socio-demographic characteristics |  |  |  |
| Age, mean (SD) | 52.0 (9.01) | 52.3 (9.94) |  |
| Female | 76.5% | 75.0% | .215 |
| Partner, yes | 87.8% | 87.9% | .905 |
| Nr. of people in household, mean (SD) | 2.7 (1.15) | 2.7 (1.17) | .844 |
| Children aged 0-12, yes | 15.7% | 16.3% | .608 |
| Educational level  Primary  Secondary  Tertiary | 28.5%  43.3%  28.2% | 27.8%  41.4%  30.8% | .125 |
| Employed, yes | 73.7% | 72.5% | .325 |
| Voluntary work, yes | 39.9% | 40.5% | .687 |
| Caregiver health |  |  |  |
| General health perception  Poor/fair  Good  Very good  Excellent | 12.8%  60.2%  22.1%  4.9% | 13.7%  59.4%  21.0%  5.9% | .291 |
| Somatisation, median (IQ range) | 16 (14-19) | 16 (14-19) | .714 |
| Caregiving situation |  |  |  |
| Caregiver cares for:  Spouse, yes (vs. no)  Parent (in-law), yes (vs. no)  Child (in-law), yes (vs. no)  Someone else, yes (vs. no) | 10.9%  64.8%  15.7%  23.2% | 11.7%  62.9%  15.5%  24.8% | .382  .168  .862  .188 |
| More than 1 care recipient, yes | 29.1% | 29.7% | .618 |
| Living together with care recipient, yes | 21.6% | 21.5% | .918 |
| Caregiving duration (years), median (IQ range) | 5 (2-10) | 4 (2-10) | .858 |
| Hours of household care tasks ^b^  0 hours  1-4 hours  4-8 hours  >8 hours | 21.2%  55.3%  11.6%  11.8% | 20.8%  54.5%  11.8%  12.9% | .713 |
| Hours of personal care tasks ^b^  0 hours  1-4 hours  4-8 hours  >8 hours | 58.8%  29.3%  6.1%  5.8% | 59.3%  29.1%  5.5%  6.0% | .800 |
| Hours of other care tasks ^b^  0 hours  1-4 hours  4-8 hours  >8 hours | 8.2%  68.8%  14.4%  8.5% | 9.4%  67.9%  13.8%  8.9% | .438 |
| Caregiving outcomes |  |  |  |
| Caregiver burden, median (IQ range) | 20 (5-50) | 20 (5-50) | .761 |
| Caregiver satisfaction, median (IQ range) | 80 (50-90) | 80 (50-90) | .680 |
| ^a^ Number of respondents might vary between variables due to item non-response.  ^b^ Item non-response for hours of household care, personal care, and other care was imputed using multiple imputation.  ^c^ Chi-square test is reported for all variables, except for age and nr. of people in household (Independent Sample T-test) and for somatization, caregiving duration, caregiver burden, and caregiver satisfaction (Mann-Whitney test) | | | |

Additional file 1: Characteristics of consent caregivers receiving and consent caregivers not

receiving informal care questionnaire
